# Inflammatory Cytokines, Adipocytokines, and Th17/Treg Balance in Patients with Nonalcoholic Fatty Liver Disease following Administration of Dahuang Zhechong Pills

**DOI:** 10.1155/2022/8560831

**Published:** 2022-01-07

**Authors:** Xiaohua Duan, Jianlin Lv, Hebei Jiang, Kefei Zheng, Yulin Chen

**Affiliations:** ^1^Medical Community of Yueqing Third People's Hospital, Yueqing City, Zhejiang Province, China; ^2^The First Affiliated Hospital of Guangxi University of Chinese Medicine, Nanning City, Guangxi Zhuang Autonomous Region, China; ^3^Guangxi School of Chinese Medicine, Nanning City 530022, Guangxi Zhuang Autonomous Region, China

## Abstract

**Objectives:**

The occurrence and development of nonalcoholic fatty liver disease (NAFLD) is related to lipid peroxidation, imbalance of inflammatory response factors, and immune function disorder. This study was conducted with the purpose of investigating the expression levels of inflammatory cytokines and adipocytokines and Th17/Treg balance in NAFLD patients treated with Dahuang Zhechong pills (DHZCPs).

**Methods:**

The study recruited 100 NAFLD patients who were then arranged into the test group and control group. Patients in the test group were treated with DHZCPs, while patients in the control group were untreated. Peripheral TH17 and Treg cells were detected by flow cytometry, and peripheral IL-17, IL-10, hs-CRP, and TNF-*α* expression levels were determined by enzyme-linked immunosorbent assay (ELISA) methods. The concentrations of ghrelin, leptin, and adiponectin were quantitatively examined.

**Results:**

The levels of TC, TG, ALT, and AST were declined but the level of HDL-C was increased in NAFLD patients treated with DHZCPs compared with untreated patients (*P* < 0.05). The ratio of Th17/Treg in NAFLD patients treated with DHZCPs was (1.52 ± 0.21), which was significantly lower than (2.39 ± 0.45) of untreated patients (*P* < 0.05). The levels of IL-17, hs-CRP, and TNF-*α* were lower, but the level of IL-10 was higher in NAFLD patients treated with DHZCPs than that in untreated patients (*P* < 0.05). The expression levels of ghrelin and adiponectin in NAFLD patients treated with DHZCPs were evidently higher than those in untreated patients (*P* < 0.01), and the expression level of leptin in NAFLD patients treated with DHZCPs was evidently lower than that in untreated patients (*P* < 0.01).

**Conclusions:**

Administration of DHZCPs regulates the immune function of NAFLD patients by keeping Th17/Treg balance and affecting the levels of inflammatory cytokines and adipocytokines.

## 1. Introduction

Nonalcoholic fatty liver disease (NAFLD) is a type of liver disease that affects approximately one-quarter of the adult population worldwide, leading to a substantial burden of ill health with wide-ranging social and economic implications [1]. NAFLD represents a progressive disease that develops from steatosis with or without mild inflammation to nonalcoholic steatohepatitis characterized by necroinflammation and faster fibrosis progression [2]. NAFLD is characterized by fat accumulation, insulin resistance, and acquired metabolic stress liver injury. It is recognized as an important cause to evolve towards cirrhosis, even hepatocellular carcinoma [3]. The occurrence and development of NAFLD is a complex process with multiple factors. In addition to lipid peroxidation, obesity, and diabetes, Hispanic ethnicity and genetic polymorphisms of GCKR, TM6SF2, PNPLA3, MBOAT7, and HSD17B13 genes are identified as risk factors of NAFLD [4]. Interestingly, the pathogenesis of NAFLD involves several immune cell-mediated inflammatory processes as well as an imbalance of immune function [5, 6]. T helper 17 (Th17) is a T-cell subset that confers a proinflammatory action and normally benefits host defense by releasing its effector cytokine, such as interleukin-17 (IL-17). Th17 cells are considered as major inducers of autoimmunity, leading to tissue inflammation [7]. Regulatory T cells (Tregs) and CD4+ CD25+ foxp3+ phenotypes are involved in immunological self-tolerance and autoimmunity suppression, and the balance between Th17 cells and Tregs has emerged as a prominent factor in regulating autoimmunity [8].

It has been reported that, up to 80% of people with obesity and more than 60% of diabetic patients had NAFLD [9]. The treatment and management of NAFLD is usually limited to pharmacological therapies and lifestyle intervention for weight loss [10, 11]. Pioglitazone, a PPAR-*γ* agonist, is an effective drug of choice to reduce progression of fibrosis in NAFLD people with diabetes [12]. Vitamin E is mainly applied to pediatric patients with NAFLD and may be considered as a treatment in adults without diabetes [13]. There have been phase III or phase IIb randomized controlled trials that were performed to test anti-inflammatory and antifibrotic agents and metabolism modulators for treating NAFLD [14].

Traditional Chinese medicine (TCM) has emerged as a promising therapeutic approach in the treatment of liver fibrosis due to its few side effects and high safety [15]. Dahuang Zhechong pills (DHZCPs) belong to a TCM ancient formula from that is composed of ground beetle, achyranthes, dried rehmannia, licorice, leech, white peony, almond, peach kernel, astragalus, grub, rhubarb, and other medicines [16]. DHZCPs are known for their function in promoting blood circulation and removing blood stasis and clearing heat clearance and dryness moisturization, as well as nourishment of Yin and blood [17]. Previous evidence demonstrated that DHZCP formula seems to inhibit expressions of serum biomarkers of liver fibrosis in patients with chronic hepatitis B [18]. More importantly, a randomized controlled clinical trial was conducted to investigate the efficacy and safety of DHZCPs in treating patients with silicosis, suggesting DHZCPs could improve the lung function, the quality of life, and the exercise capacity of silicosis patients [19]. In this study, we used DHZCPs to treat NAFLD and observe its clinical efficacy and its regulation on inflammatory cytokines and adipocytokines and Th17/Treg balance in patients with NAFLD treated with DHZCPs.

## 2. Materials and Methods

### 2.1. Study Participants

This study included 100 NAFLD patients diagnosed and treated in our hospital from January 2018 to January 2020. It contained 62 males and 38 females, who aged 24 to 68 years, with an average of (52.36 ± 9.64) years. The NAFLD patients were confirmed by the diagnostic criteria of NAFLD [20]. Those patients should be excluded if they had administration of immunomodulators within the past 1 month and onset toxicity, drug-induced diseases or autoimmune hepatitis, hepatolenticular degeneration, hypo-*β* lipoproteinemia, congenital lipid atrophy, celiac disease, malignancies, and other specific diseases that can lead to fatty liver disease. Among them, 60 patients were assigned to the test group, and the remaining 40 patients were considered as the control group. Written informed consent was obtained from each participant or their guardians. The study protocol was approved by the Ethics Committee of The First Affiliated Hospital of Zhengzhou University.

### 2.2. Study Design

All study participants were asked to change their lifestyle, such as dieting, moderate exercise, smoking and alcohol cessation and control the deterioration of primary disease, underlying disease, and accompanying diseases so as to reduce the fat content in the liver and promote fat regression. The patients in the test group received oral administration of DHZCPs three times a day (5.0 g per day) for 3 months. All patients were given a low-fat and low-cholesterol diet, namely, intake of total calories comprising 50%–60% carbohydrate, 10%–20% protein, and no more than 30% fat. The ratio of saturated fatty acid, monounsaturated fatty acid, and polyunsaturated fatty acid was 1 : 1 : 1.

### 2.3. Flow Cytometric Analysis of TH17 Cells and Tregs

Fasting venous blood (5 mL) was collected from each subject, placed into anticoagulated tubes, and added with hemolysin. The mixture was centrifuged and added with lymphocyte separation solution. After centrifugation at 1000 ×g for 20 min, the lymphocyte layer was extracted. The cell concentration was adjusted to 1 × 10^5^ cells/mL in phosphate buffered saline (PBS). Th17 cells were immunophenotyped as CD4+IL-17+ and Tregs as CD4+APC-CD25+PE-Foxp3+ [21] by using an FACSCalibur flow cytometer (Beckman Coulter, USA) using FITC-CD4, APC-CD25, PE-Foxp3, and APC-IL-17 fluorescent antibodies (eBioscience, USA). Further flow cytometric analysis was performed using the CelQuestv3.2 analysis software.

### 2.4. Detection of IL-17, IL-10, hs-CRP, and TNF-*α*

Fasting venous blood (2 mL) was collected from each subject, placed into anticoagulated tubes, and centrifuged at 1000 ×g for 10 min to obtain the serum. The levels of IL-17, IL-10, and TNF-*α* were detected by the enzyme linked immunosorbent assay (ELISA) method, strictly following the instructions provided by the kits (Nanjing Jiancheng Institute of Bioengineering, China). The absorbance value of each well at a wavelength of 450 nm was obtained using a microplate reader (Bio-Rad, USA), normalizing to the standard curve. The level of hs-CRP was measured with a high-sensitivity immunoturbidimetric assay (Roche Diagnostics, Indianapolis, IN, USA) using an automated clinical chemistry analyzer.

### 2.5. Measurement of Ghrelin, Leptin, and Adiponectin Levels

The concentration of leptin was quantitatively examined by enzyme amplified sensitivity immunoassay (BIOSOURCE, Invitrogen, USA). Referrals were expected to range from 0.5 to 41.5 ng/ml according to the supplier. The concentrations of ghrelin were analyzed by radioimmunoassay (BIOSOURCE, Invitrogen). The expected concentrations according to the producer were between 300 and 4,000 pg/ml. The concentration of adiponectin was determined by using the Human Adiponectin RIA Kit (Linco Research Inc., St Charles, USA) in accordance with the instruction provided by the supplier.

### 2.6. Statistical Processing

The SPSS19.0 statistical software was used to perform statistical processing on collected data. The comparison between groups was made using an independent *t*-test, the comparison within groups was performed using a paired *t*-test, and the comparison among multiple groups was carried out using one-way analysis of variance. The count data were analyzed using the chi-square test. *P* < 0.05 was considered as statistically significant.

## 3. Results

### 3.1. Improved Liver Function of NAFLD Patients after Treatment with DHZCPs

The levels of TC, TG, HDL-C, ALT, and AST were determined to evaluate the liver function of NAFLD patients after treatment with DHZCPs. As shown in Table 1, it was found that the levels of TC, TG, ALT, and AST were declined in NAFLD patients treated with DHZCPs, which were lower than those in untreated NAFLD patients (*P* < 0.05). The level of HDL-C was increased NAFLD patients treated with DHZCPs, which is higher than that in untreated NAFLD patients (*P* < 0.05).

### 3.2. A Lower Ratio of Th17/Treg Cells in NAFLD Patients Treated with DHZCPs

Th17 and Treg, two subsets of CD4+ T helper cells, seem to maintain a subtle balance for organic immune homeostasis including liver. The disturbance of Th17/Treg balance in liver has been found to be associated with hepatic injury and disease. Accordingly, we performed flow cytometric analysis of peripheral TH17 and Treg cells in NAFLD patients treated with DHZCPs and untreated NAFLD patients. The proportion of Th17 cells in the test group was (11.23 ± 1.39) %, which was notably lower than the (14.36 ± 2.28) % of the control group (*P* < 0.05). The proportion of Treg cells in the test group was (7.36 ± 1.01) %, which was remarkably higher than the (6.01 ± 0.89) % of the control group (*P* < 0.05). The ratio of Th17/Treg cells in the test group was (1.52 ± 0.21), which was significantly lower than the (2.39 ± 0.45) of the control group (*P* < 0.05, Table 2).

### 3.3. Changes of IL-17, IL-10, hs-CRP, and TNF-*α* Expressions in NAFLD Patients after Treatment with DHZCPs

The serum levels of IL-17, IL-10, hs-CRP, and TNF-*α* were determined to evaluate the production of inflammatory cytokines in NAFLD patients after treatment with DHZCPs. It was observed that the expression levels of IL-17, hs-CRP, and TNF-*α* in the test group were remarkably lower than those in the control group (*P* < 0.01), and the expression level of IL-10 in the test group was remarkably higher than that in the control group (*P* < 0.05, Table 3).

### 3.4. Changes of Ghrelin, Leptin, and Adiponectin Expressions in NAFLD Patients after Treatment with DHZCPs

The expression levels of ghrelin, leptin, and adiponectin were determined in NAFLD patients after treatment with DHZCPs. Results showed that the expression levels of ghrelin and adiponectin in the test group were evidently higher than those in the control group (*P* < 0.01), and the expression level of leptin in the test group was evidently lower than that in the control group (*P* < 0.01, Table 4).

## 4. Discussion

NAFLD has become the primary cause of abnormal liver biomarkers in chronic liver disease and health examinations in China. The etiology and pathogenesis of NAFLD are not fully understood, which brings certain difficulties to its effective prevention and treatment [22]. Wang et al. [23] found that patients with NAFLD have a disorder of lymphocyte subsets. Increased inflammatory activity of CD4+ T lymphocytes plays a key role in the immune dysfunction of patients with NAFLD. Th17 and Treg cells are newly discovered members of CD4+ T lymphocytes. Th17 plays an anti-inflammatory role mainly secreting the inflammatory factor IL-17 Treg cells regulate the expression of IL-17 mainly through secreting IL-10 inflammatory inhibitory factor. Therefore, there is a dynamic balance between Th17/Treg cells and secreted IL-17/IL-10 factors [24].

Th17/Treg imbalance is involved in the occurrence of many diseases. He et al. [25] found abnormal expression of Treg and Th17 cells in patients with NAFLD. Rolla et al. [26] found that the expression of Th17 cells is very important for the occurrence of NASH and the development of fibrosis in NAFLD mice. The present study found that the proportion of Th17 cells and the ratio of Th17/Treg in patients with NAFLD were significantly higher than those in the control group. The effector molecules IL-17 and IL-17/IL-10 ratios were also significantly higher than those in the control group. The abovementioned results all indicate that Th17/Treg imbalance is involved in the pathogenesis of NAFLD. In this study, relevant tests results were compared in the test group (treated with DHZCPs) and the control group (untreated). It was found that the proportion of TH17 cells in the test group was lower than that of the control group. In addition, the proportion of Treg cells in the test group was higher than that of the control group. The ratio of Th17/Treg cells in the test group was lower than that in the control group.

Accumulating evidence shows that adipocytokines are involved in insulin resistance, leading to the occurrence of NAFLD [27]. Fat, as a highly active tissue, stores heat in the form of triglycerides and secretes a series of protein hormone-like factors such as leptin, adiponectin, resistin, and visfatin [28]. Many adipocytokines were associated with the inflammation and immune regulation [29]. Furthermore, they regulate the metabolism of the body as a manner of endocrine, paracrine, and autocrine [30]. Leptin can regulate body fat and energy balance. A study found that leptin in some patients with NAFLD was significantly increased, suggesting that leptin resistance may exist in these patients [31]. High leptin level and leptin resistance can increase insulin level, promote insulin resistance, affect the insulin signal transduction pathway of hepatocytes, and increase fatty acid content in hepatocytes, resulting in fatty liver occurrence [32]. TNF-*α* is the main cytokine causing liver damage in NAFLD [33]. Although the 3D structure of TNF-*α* is very similar to that of adiponectin, their functions are completely opposite. The decrease of adiponectin level in NAFLD patients can lead to the increase of fatty acid synthesis, the accumulation of TG, and the obstruction of fatty acid oxidation [34]. Ghrelin is a ligand of endogenous growth hormone secretagogue receptor found in recent years. Ghrelin not only promotes the secretion of growth hormone but also increases body weight and regulates energy metabolism. It was found that ghrelin is closely related to insulin, glucose, and lipid metabolism [35]. A low ghrelin level is recognized as a risk factor for type II diabetes and impaired glucose tolerance [36]. Ghrelin modulates the central appetite regulatory network, especially neuropeptide *γ*, and ghrelin interacts with a variety of adipocytokines, such as leptin and insulin, and plays an important role in the pathological process of obesity. As shown by our data, administration of DHZCPs significantly increased the expression levels of ghrelin and adiponectin but decreased the expression level of leptin in NAFLD patients treated with DHZCPs. These findings were supported by previous studies.

In summary, the abovementioned research results show that the administration of DHZCPs regulates the immune function of NAFLD patients by keeping Th17/Treg balance and affecting the levels of inflammatory cytokines, IL-17, IL-10, hs-CRP, and TNF-*α*, and adipocytokines, leptin, adiponectin, and ghrelin.

## Figures and Tables

**Table 1:** The serum levels of TC, TG, HDL-C, ALT, and AST between the test and control groups.

Group	TC (mmol/L)	TG (mmol/L)	HDL-C (mmol/L)	ALT (U/L)	AST (U/L)
Test group (*n* = 60)	3.65 ± 0.82^*∗*^	1.31 ± 0.39^*∗*^	1.45 ± 0.43^*∗*^	21.56 ± 6.52^*∗∗*^	24.28 ± 6.88^*∗∗*^
Control group (*n* = 40)	5.56 ± 074	2.76 ± 0.38	1.26 ± 0.53	63.58 ± 8.68	57.69 ± 8.02

^*∗*^ *P* < 0.01  and ^*∗∗*^*P* < 0.05 compared with the control group.

**Table 2:** The proportion of TH17 cells, Treg cells, and their ratio between the test and control groups.

Group	TH17 (%)	Treg (%)	Th17/Treg
Test group (*n* = 60)	11.23 ± 1.39^*∗*^	7.36 ± 1.01^*∗*^	1.52 ± 0.21^*∗*^
Control group (*n* = 40)	14.36 ± 2.28	6.01 ± 0.89	2.39 ± 0.45

^*∗*^ *P* < 0.01 compared with the control group.

**Table 3:** The serum levels of IL-17, IL-10, hs-CRP, and TNF-*α* between the test and control groups.

Group	IL-17 (*μ*g/L)	IL-10 (*μ*g/L)	hs-CRP (mg/L)	TNF-*α* (ng/L)
Test group (*n* = 60)	13.52 ± 2.11^*∗*^	8.46 ± 1.12^*∗∗*^	0.87 ± 0.41^*∗*^	6.73 ± 2.01^*∗∗*^
Control group (*n* = 40)	21.58 ± 3.56	6.11 ± 1.01	1.13 ± 0.37	12.20 ± 2.56

^*∗*^ *P* < 0.01  and ^*∗∗*^*P* < 0.05 compared with the control group.

**Table 4:** The serum levels of ghrelin, leptin, and adiponectin between the test and control groups.

Group	Ghrelin (ng/L)	Leptin (ng/L)	Adiponectin (ng/L)
Test group (*n* = 60)	7.33 ± 2.32^*∗*^	7.49 ± 2.12^*∗∗*^	12.78 ± 6.57^*∗∗*^
Control group (*n* = 40)	4.65 ± 1.72	16.05 ± 5.42	6.83 ± 3.33

^*∗*^ *P* < 0.01  and ^*∗∗*^*P* < 0.05 compared with the control group.

## Data Availability

The data used to support the findings of this study are included within the article.
